# Supplementary material for: Crossover interference mediates multiscale patterning along meiotic chromosomes
Source: Nat Commun. 2025 Nov 25;16:10453. doi: 10.1038/s41467-025-65423-6 (PMC12647850; doi:10.1038/s41467-025-65423-6)
Supplement: Supplementary file 1 — Supplementary Information [file 41467_2025_65423_MOESM1_ESM.pdf]

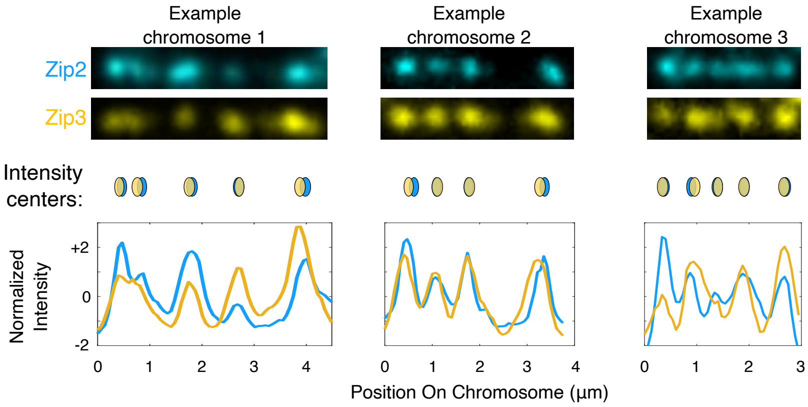

Supplementary Fig. 1. Co-localization of intensity centers of crossover associated proteins Zip2 and Zip3. The positions of canonical crossovers have been defined, and their patterns analyzed, by the disposition of fluorescent foci of both Zip2 and Zip3 along the synaptonemal complexes of budding yeast pachytene chromosomes. *Top*, micrographs of three example spread pachytene chromosomes immunostained for both Zip2 and Zip3 proteins, and *bottom*, their corresponding normalized signal intensity profiles. Normalized intensity is defined as the number of standard deviations from the mean for each signal (blue Zip2, yellow Zip3). Intensity centers of Zip2 (blue) and Zip3 (yellow), as defined by peaks in normalized signal intensity, show significant co-localization (*middle*). The two signals mark the same sites, as shown previously[1]. Minor differences in signal shapes and the positions of intensity centers, are all less than 135 nm, well within the range expected for the known sizes and differences in orientation and/or state dynamics of canonical crossover complexes (e.g.[2]).

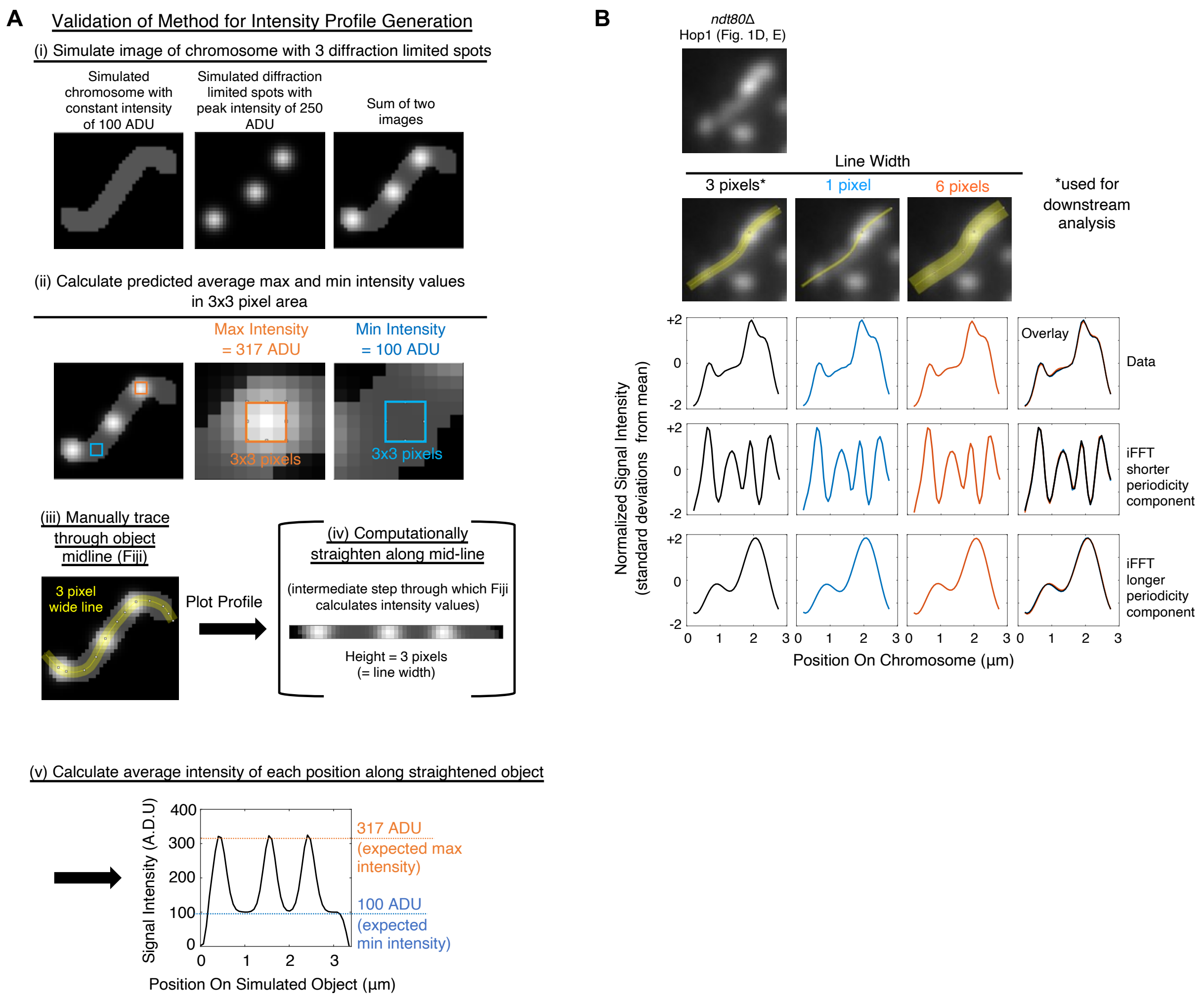

Supplementary Fig. 2. Validation of Fiji methodology for generating signal intensity profiles. (**A**) (i) A chromosome-like object was simulated as a curvilinear object of flat pixel intensity (100 analog-to-digital units, 'ADU') overlaid with three diffraction limited spots of known peak pixel intensity value (250 ADU). (ii) Minimum and maximum signal intensity values along this object were determined as the average pixel intensities within regions of interest (ROIs) measuring 3x3 pixels. For positions of spots, the ROI was centered on the pixel of brightest intensity; for positions lacking spots, the ROI was centered on a region along the object midline that was well-separated from adjacent spots. (iii) The path of the chromosome-like object was traced in Fiji using a 3-pixel wide line and the corresponding signal intensity values extracted using the Plot Profile function of Fiji. (iv) This function works by computationally straightening the traced line and then calculating the average intensity value of each column of pixels on the straightened image (i.e. at each position along the straightened image). (v) This approach faithfully recapitulated the shape of the 1D-signal of the simulated object with the pre-determined minimum and maximum intensity values. (**B**) All intensity profiles analyzed in the Results section were obtained using a 3-pixel wide trace line with the corresponding signal

intensity values extracted using the Plot Profile function of Fiji as described in (A). The 3-pixel width was selected to capture the majority of the chromosome signal with a minimum contribution of extra-chromosomal pixels lacking signal. However, control analysis shows that signal intensity profiles obtained in this way do not depend on the thickness of the trace line. This outcome is illustrated for the Hop1 signal of the example wild type chromosome shown in text Fig. 1D - F (top). Indistinguishable results are obtained for line thicknesses of 1, 3 and 6 pixels, as shown individually in middle left and middle right panels and their overlay in the right panels. This robustness reflects the fact that signal intensity values are symmetrically distributed around the midline trace.

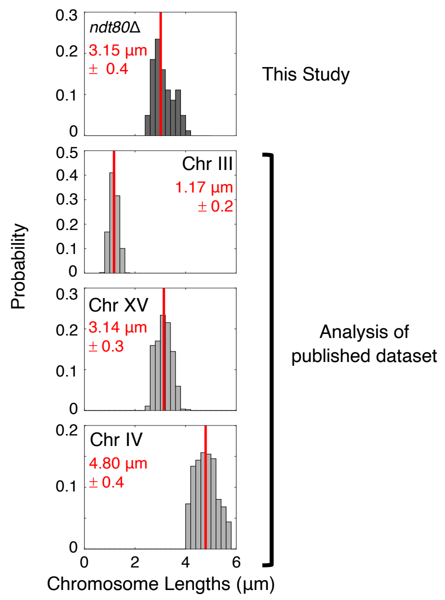

Supplementary Fig. 3. Measured chromosomes are narrowly distributed around 3.2 µm in length, similar to those expected for chromosome XV. Comparison of the distribution of lengths of measured wild type (*ndt80*Δ) chromosomes in this study (top, n = 81), with distributions of lengths of chromosome (Chr) III, XV and IV (n = 683, 822 and 410 chromosomes respectively; data from[3]). Red lines indicate mean lengths. Red values show mean lengths ± one standard deviation.

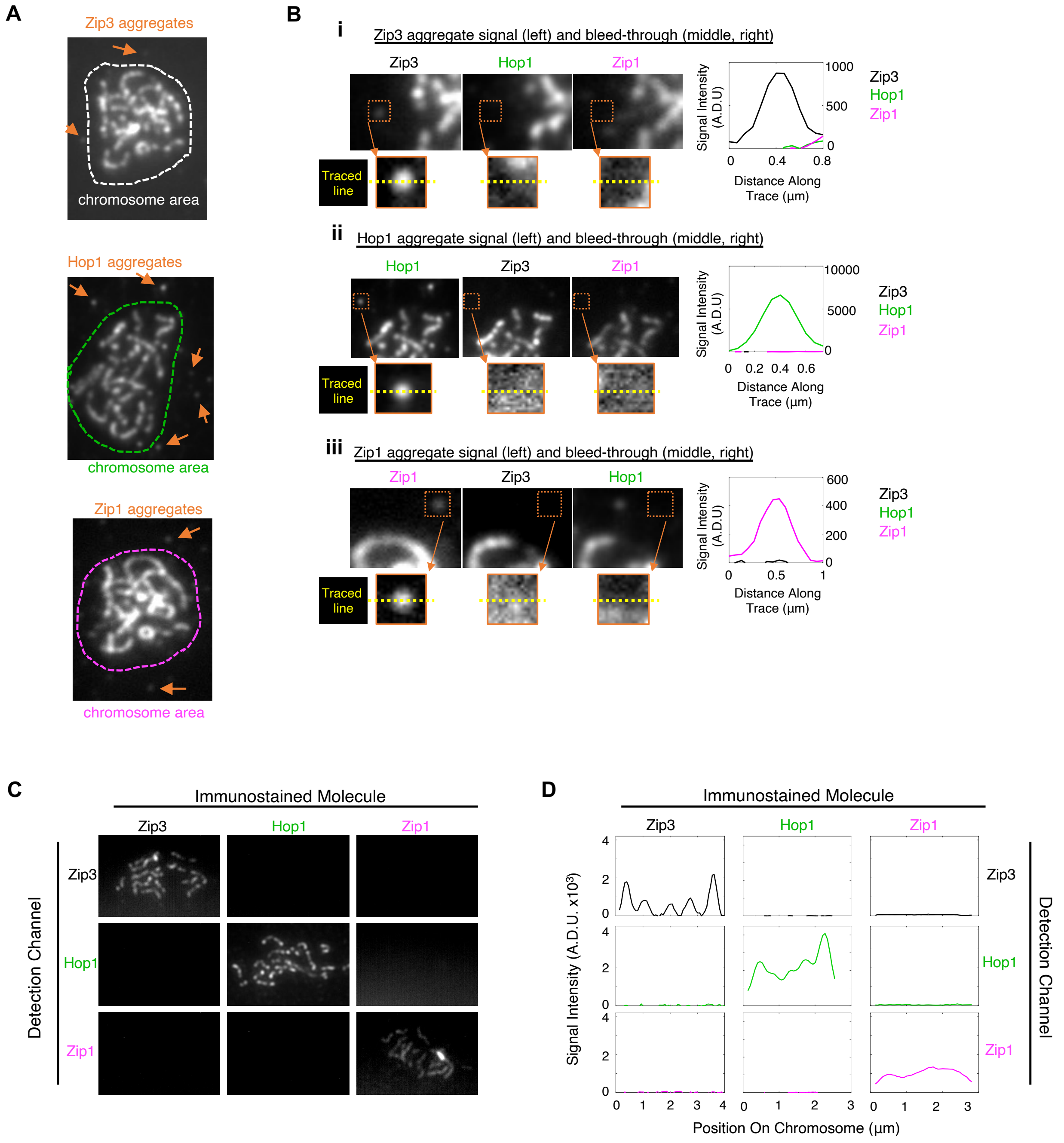

Supplementary Fig. 4. Spectral bleed-through is negligible. We used two different approaches to assess the extent of spectral bleed-through from each immunostained molecule into the detection channels for the other two immunostained molecules. (**A**, **B**) In a first approach, we were able to assess bleed-through for each molecule in actual experimental samples that were immunostained for all three molecules. In such samples, each of the secondary antibodies accumulates in non-chromosome-associated aggregates, seen as puncta outside the chromosome area (**A**, *ndt80*Δ pachytene chromosomes; aggregates indicated by arrows; dashed

lines indicate chromosome areas). The non-chromosome-associated aggregates of the three proteins of interest (Zip3, Hop1 and Zip1) do not co-localize. Bleed-through was assessed by quantifying the signal intensity for each type of aggregate in each of the three analysis channels. For this purpose, a trace was made through the puncta midlines in Fiji (**B**, left panels; yellow lines). The corresponding signal intensity values in all three imaging channels were then extracted and quantified using the Plot Profile function of Fiji (**B**, right). Bleed-through is negligible in all cases. (**C**, **D**) In a second approach, spectral bleed-through was examined in single-molecule immunostaining experiments, where spread chromosomes were immunostained against either Zip3, Hop1, or Zip1 ('Immunostained Molecule') and then imaged by widefield fluorescence microscopy under the same conditions, and in the same three "detection" channels (i.e. excitation and emission wavelengths), used for the triple-target immunostaining experiments used in this study and in (A, B) above ('Detection Channel'). (**C**) Micrographs of spread chromosomes that were isolated from pachytene arrested wild type cells and immunostained individually for Zip3 (*left*), Hop1 (*middle*) or Zip1 (*right*). Visually apparent signals are only observed in the expected detection channels. (**D**) Visual impressions were confirmed by defining signal intensity profiles of an individual chromosome from each of the images presented in (C). Spectral bleed-through is negligible in all cases. A.D.U., analog-to-digital units.

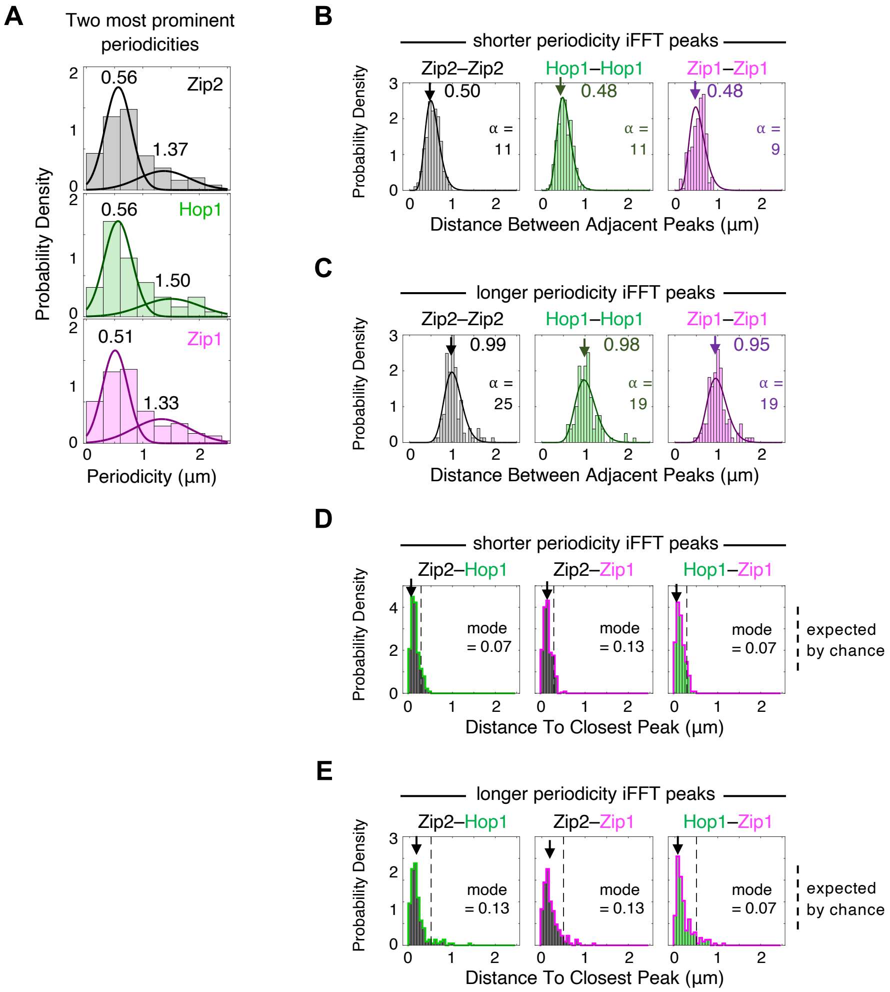

Supplementary Fig. 5. Fourier transform analysis defines two groups of intensity signals, of shorter and longer periodicities, whose peaks occur in triads, *extended*. Analysis of Zip2, Hop1 and Zip1 signal intensity profiles along pachytene chromosomes isolated from a wild type strain in the absence of pachytene arrest (*NDT80*), with corresponding L_{coc} values (n = 56 chromosomes). (**A**) As in Fig. 2C. Distributions of the two highest amplitude peaks in the FFT amplitude spectra, across all measured chromosomes. Solid lines show best fit two-component Gaussian mixture models with their corresponding means, consistent with two groups of shorter and longer periodicity. (**B** and **C**, as in Fig. 3C and D) distributions of distances between adjacent shorter (**B**) and longer (**C**) periodicity iFFT peaks, with best fit gamma distributions (solid lines) and their corresponding modes (arrows), and shape parameters (α). (**D** and **E**, as in Fig. 3E and F) distributions of distances between each Zip2 peak and its closest Hop1 peak and analogous distributions for Zip2 – Zip1 and Hop1 – Zip1, for shorter (**D**) and longer (**E**) periodicity iFFT peaks. Dashed lines indicate medians expected if the two molecules were independently phased (0.27 – 0.28 µm and 0.55 – 0.57 µm for all pairwise combinations of

molecules; dashed lines). Note that all results are indistinguishable for Zip2 (this figure) and Zip3 (text Fig. 3C – F).

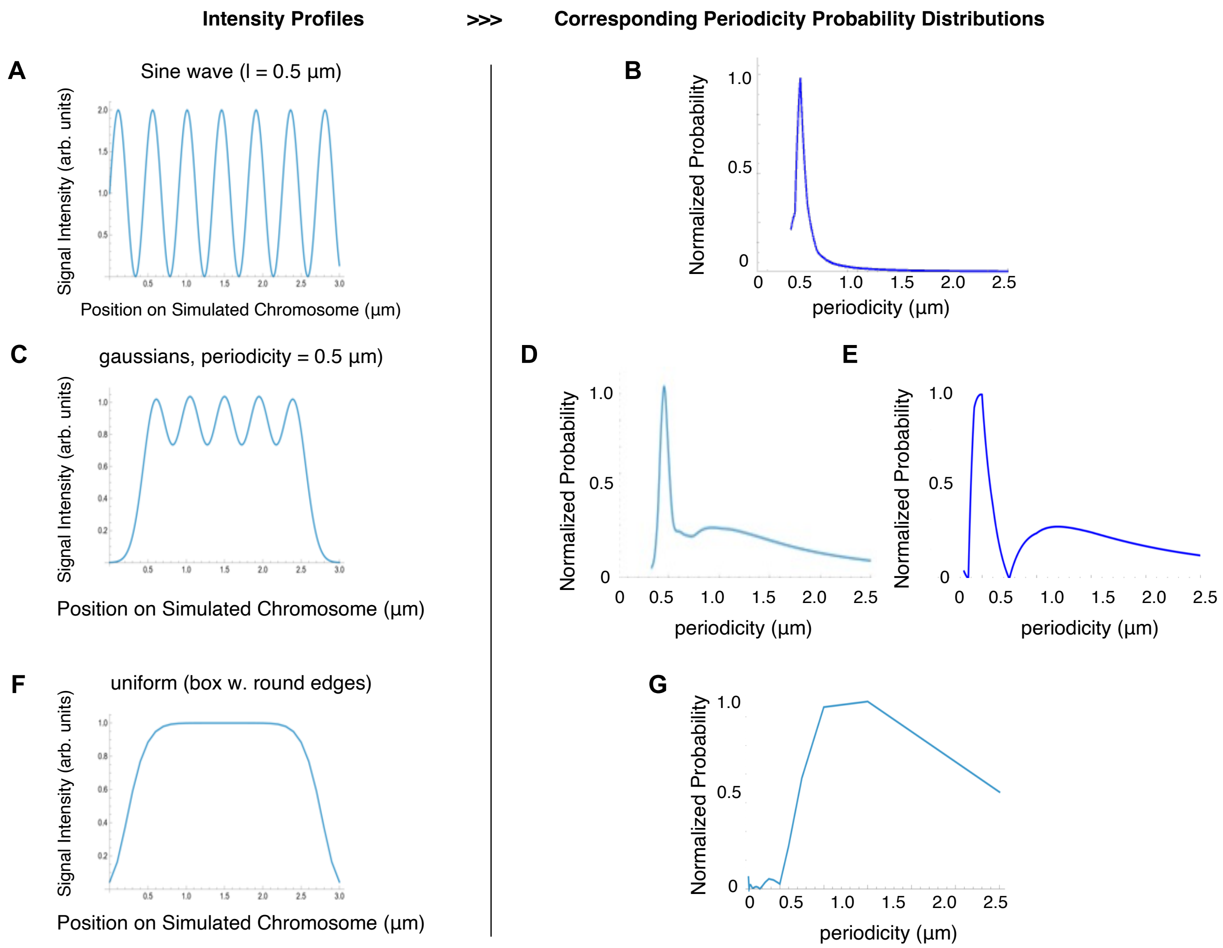

Continued on next page.

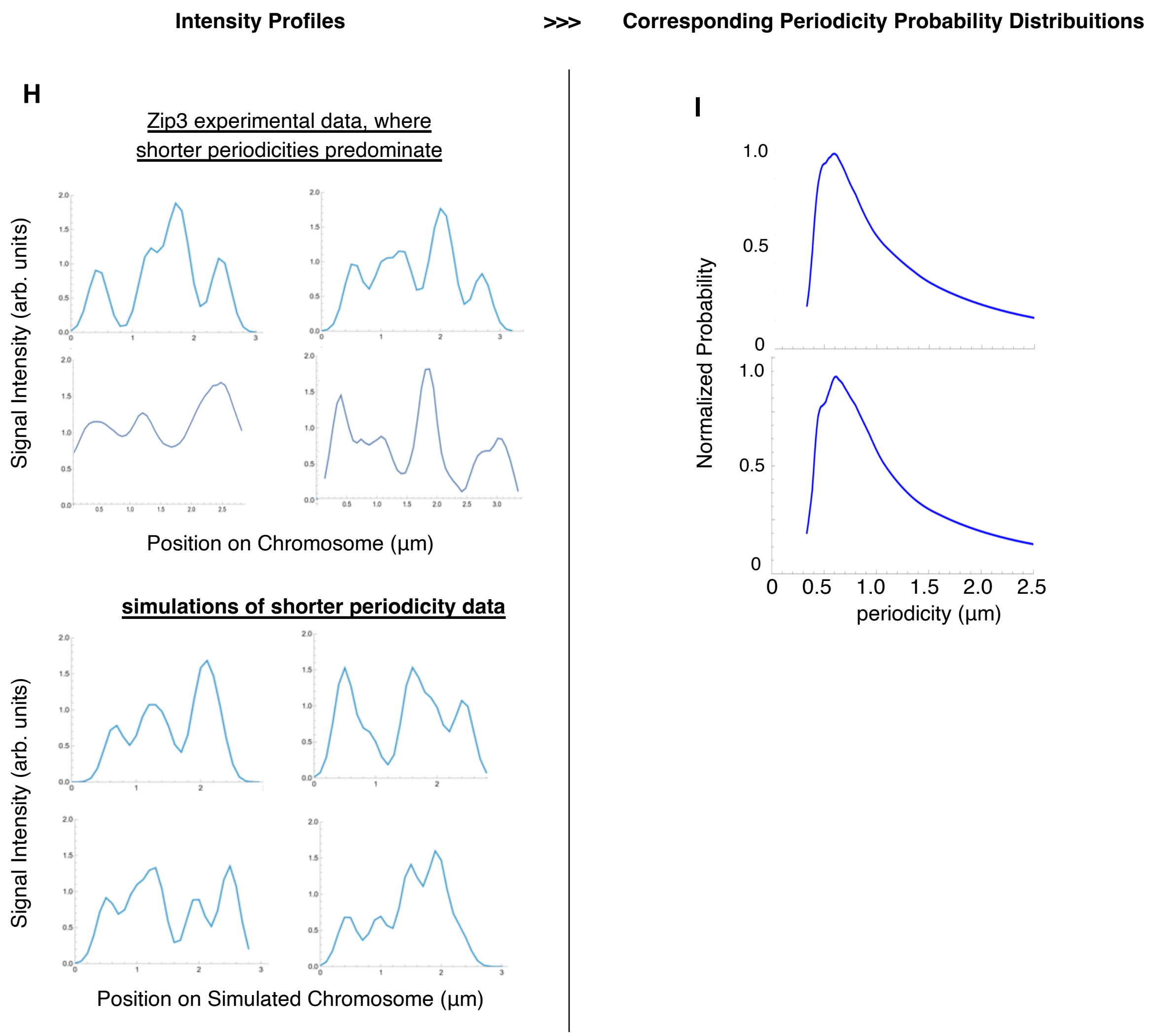

Supplementary Fig. 6. Deconvolution of total FFT outputs into two groups of heterogeneous shorter and longer periodicity signals, of ~0.5 μm and ~1 μm. For each of the three examined molecules, the sum of the FFT outputs for all chromosomes, exhibit the patterns expected from the analysis of "two-most-prominent peaks": two peaks at values for two component heterogeneous groups signals, of ~0.5 μm and ~1 μm (Hop1 and Zip1) or, for Zip3, a complex peak which can be understood as reflecting the same two groups but with much higher abundance of the lower periodicity signal as compared to the higher periodicity signal (text Fig. 2D, reproduced below as **Panel J** top).

By further analysis, we can deconvolve each of the three total signals into the two component periodicity groups, with appropriate relative abundancies. To do this, we first simulated distributions of shorter periodicity signals (whose existence and nature are clear from the correspondence to canonical crossovers, text). We then took the Fourier transforms of 100 simulated cases, summed those outputs, and subtracted that outcome from the sum of experimental periodicities. We then asked whether significant (longer) periodicities remain

after subtraction. If longer periodicities are present in the data, the residuum would exhibit a corresponding broad peak centered on ~1 µm. The outcome, for each of the three molecules, is a clear peak at the expected ~1 µm periodicity (e.g. grey dashed line, text Fig. 2E, reproduced in **Panel J** below).

Simulations. This analysis involved a series of control experiments as well as simulations of the experimental data set. Several different types of signal intensity profiles were simulated (left panels: **A**, **C**, **F**, and **H bottom**). For each type, 100 individual simulated profiles were generated, summed in k-vector space, and converted to position space, just as for the experimental data (Methods) (right panels: **B**, **D**, **E**, **G**, **I**).

(1) We first simulated a sine wave of wavelength 0.5 µm (**A**). Fourier transforms for this case do not show any longer-periodicity peaks, whether the signal is confined to 3 µm (i.e. the length of an analyzed chromosome) (**B**) or not confined (not shown). This result confirms that our analysis algorithms are working correctly.

(2) We next analyzed a signal comprising Gaussian peaks separated by exactly 0.5 µm along an object of exactly 3 µm (**C**). In this case, the Fourier transform exhibits significant longer periodicities (**D**), despite the fact that no such periodicities are present in the data. Inclusion of variation in chromosome length, as for experimental data (~3.15 $\pm$ 0.4 µm; Supplementary Fig. 3) smooths the curve somewhat (**E**) but does not alter the basic result (**D** versus **E**). These results illustrate that confinement of Gaussians of a given periodicity in a confined space can lead to emergence of aberrant longer periodicities in Fourier transform outputs. This effect can also be seen by taking the Fourier transform of a uniform intensity distribution along an object of 3 µm, modeled as a square with smooth edges (**F**). The result is a broad long wavelength peak with very little contribution from short wavelengths (**G**).

(3) Finally, we simulated signal intensity profiles that closely match the actual experimental data. The signals in this situation comprised Gaussian peaks separated by an average of ~0.5 µm, arrayed along a chromosome of ~3 µm length. Three types of variations were included. (i) We introduced heterogeneity in inter-peak distances corresponding to that observed by peak-finding analysis of primary data (**H top**) (and given by the known distribution distances between Zip3 foci in standard analysis (text). (ii) Peak heights were varied in correspondence to variations observed by visual inspection of primary data (**H top**). And (iii) chromosome lengths were varied as occurs in experimental data (Supplementary Fig. 3). Additionally, the density of data points in the simulation was 0.1 µm, close to the 0.07 µm of experimental data (text)). Examples of individual simulations are shown in (**H bottom**). The sum of the Fourier transforms for such sets of simulated data give a single broad, smooth peak with substantial probabilities of

longer periodicities (two examples in **I**). We note that longer periodicities are significantly more prominent in this simulated data than for simulations of similarly-confined Gaussian peaks that are perfectly homogeneous in spacing and peak height (**H**, **I** versus **C – E**). Heterogeneity in spacing in the simulated data is especially important for this difference. We also note that this exercise was performed for 100 chromosome simulations, with slightly varying values of relevant parameters in different cases. The same basic result was obtained in all cases, implying that the basic output (and thus the conclusions from subtraction analysis below) are robust to variations in parameter values.

Subtraction of simulated data-matched shorter periodicity probabilities from total experimental probabilities. To evaluate whether experimental data exhibited longer periodicities over and beyond those expected to occur from the shorter periodicities alone, the periodicity distribution for simulation (3) was subtracted from that of the total experimental distribution, for each molecule. Three features of the outcome strongly support the presence of two major groups of periodicities, longer as well as shorter, for all three molecules (text Fig. 2E; reproduced in **Panel J** below).

- First, in the residuum after subtraction (grey dashed line), remaining periodicities rise into a broad peak centered at ~1 µm (grey dashed line; red arrows). This feature shows that a group of longer periodicities, of ~1 µm, is present in the experimental data. (That is: longer periodicities are present in the actual data, beyond those that appear artifactually due to confinement of ~0.5 µm periodicities to a ~3 µm object as described above). Longer periodicities that are present in the data but not in the simulation are indicated by the distance spanned by gold arrows).

- Second, in the residuum after subtraction (grey dashed line), the distribution of periodicity probabilities falls to zero, or near zero, at ~0.5 µm (blue arrows). Thus, the simulated shorter periodicity distribution accounts for essentially all of the shorter periodicities in the experimental data.

- Third, the level of residual longer periodicities (grey dashed line) is lower for Zip3 than for Hop1 and Zip1. This matches the fact that, in primary images, broader (longer periodicity) signals are much less prominent than narrower (shorter periodicity) signals for Zip3 as compared to the other two molecules (text).

We note that, for purposes of subtraction, the probability distribution for the simulation was normalized such that the highest value is 0.93. This gives probabilities at 0.5 µm optimally close to zero for all three molecules (and, for Zip3, to exactly zero). Normalization to 1 increased these values but did not significantly affect the fundamental finding of longer periodicities.

J

Fig. 2D

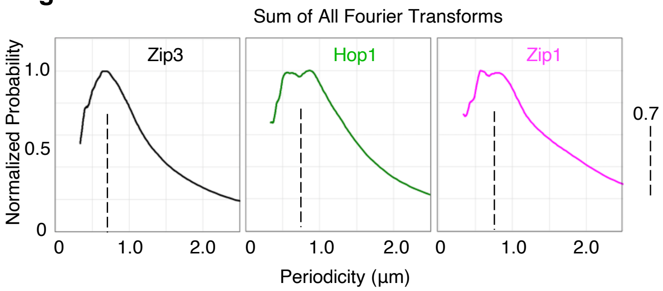

Fig. 2E

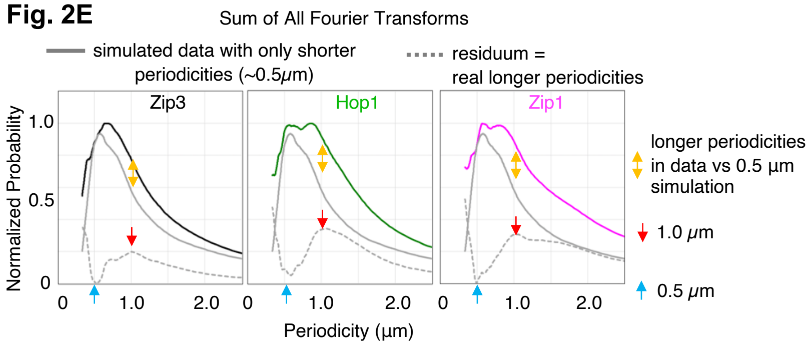

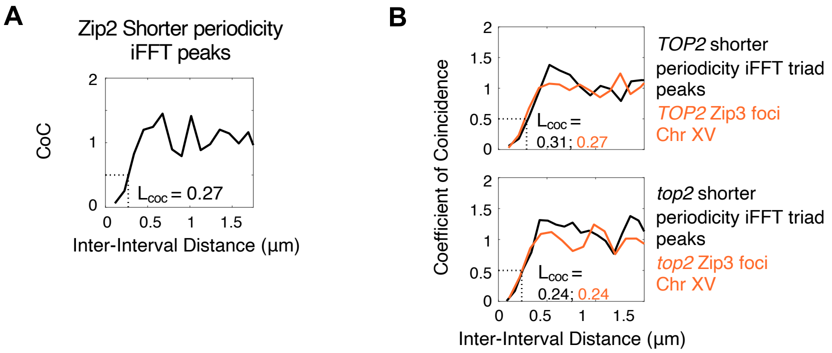

Supplementary Fig. 7. Focal triads arise at sites of "canonical" crossovers, *extension 1*. (**A**) analogous to text Fig. 4C top row. Coefficient of coincidence (CoC) curve for the Zip2 shorter periodicity iFFT peaks of pachytene chromosomes isolated from a wild type strain in the absence of pachytene arrest (*NDT80*), with corresponding L_{coc} values (n = 56 chromosomes). Note that results are indistinguishable for Zip2 (this figure) and Zip3 shorter periodicity iFFT peaks (text Fig. 4C top left). (**B**) *top2* conditions confer the same reduction in L_{CoC}, relative to wild type *TOP2* conditions for shorter periodicity triad peaks (this work; black lines) as previously shown by standard analysis of canonical crossovers (Zip3 foci; data from[3]; orange lines). 'Chr', chromosome. n = 81, 822, 60, and 175 chromosomes for *TOP2* shorter periodicity iFFT triad peaks (a reproduction of data shown in text Fig. 4C top right), *TOP2* Zip3 foci ChrXV, *top2* shorter periodicity iFFT triad peaks (a reproduction of data shown in text Fig. 4C middle right), and *top2* Zip3 foci Chr XV respectively.

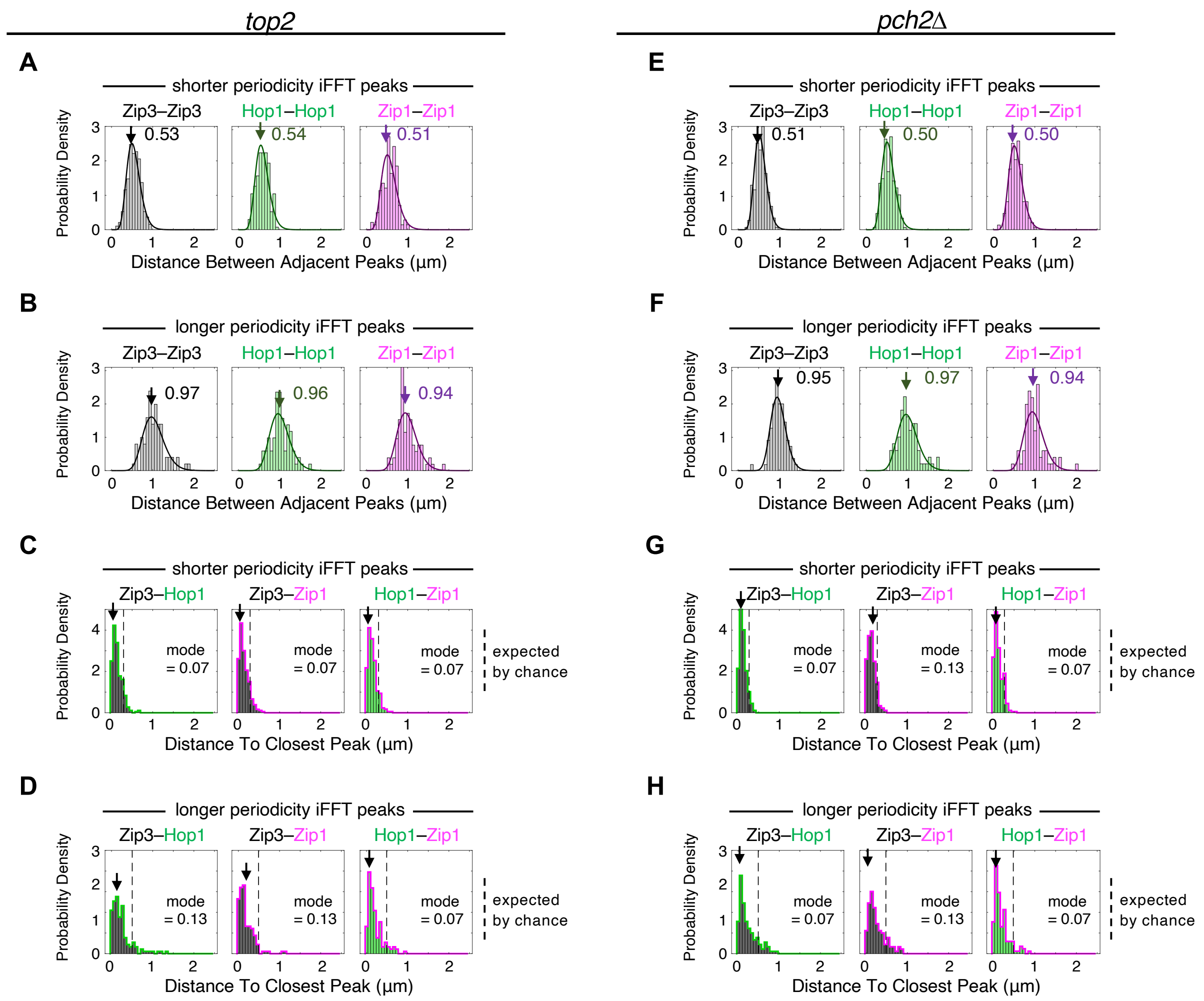

Supplementary Fig. 8. Shorter and longer periodicity triads form in the absence of Top2 and Pch2. Analysis of iFFT-defined peaks along pachytene chromosomes isolated from either cells depleted for Topoisomerase 2 (**A** – **D**; *ndt80Δ pCLB2-TOP2*, '*top2*'; n = 60) or lacking the *pch2* gene (**E** – **H**; *ndt80Δ pch2Δ*, '*pch2Δ*'; n = 52), indicates that shorter and longer periodicity triads occur in both mutant conditions. (i) the distance between adjacent peaks is the same for all three molecules (**A**, **B**, **E**, **F**); and (ii) for both shorter and longer periodicity iFFT peaks in both mutant conditions, distances between each peak and the nearest peak of a different molecule are very small (~0.07 – 0.13 μm), much smaller than expected by chance (~0.3 μm and ~0.5 μm; dashed lines), for all three pairs of molecules (**C**, **D**, **G**, **H**). (**A**, **E**) analogous to text Fig. 3C. Distance between adjacent shorter periodicity iFFT peaks with best fit gamma distribution (solid lines) and associated modes (arrows and values) indicated. (**B**, **F**) analogous to text Fig.3D. Distance between adjacent longer periodicity iFFT peaks with best fit gamma distribution (solid lines) and associated modes (arrows and values) indicated. (**C**, **G**) analogous to text Fig. 3E. Distributions of distances between each shorter periodicity iFFT Zip3 peak and its closest shorter periodicity iFFT Hop1 peak, with analogous distributions for Zip3-Zip1 and Hop1-Zip1.

Dashed lines indicate expected medians if the two molecules were independently phased (0.28 – 0.29 µm and 0.27 µm for all pairwise combinations of molecules in *top2* and *pch2* respectively). (**D**, **H**) analogous to text Fig. 3F. Distributions of distances between each longer periodicity iFFT Zip3 peak and its closest longer periodicity iFFT Hop1 peak, with analogous distributions for Zip3-Zip1 and Hop1-Zip1. Dashed lines indicate expected medians if the two molecules were independently phased (0.49 – 0.50 µm and 0.48 – 0.49 µm for all pairwise combinations of molecules in *top2* and *pch2* respectively).

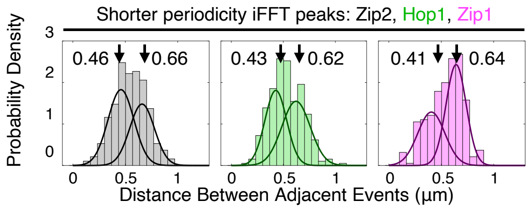

Supplementary Fig. 9. Focal triads arise at sites of "canonical" crossovers, *extension 2*. Analogous to text Fig. 4E *bottom*. Distributions of distances between adjacent shorter periodicity Zip2, Hop1 and Zip1 peaks along pachytene chromosomes isolated from a wild type strain in the absence of pachytene arrest (*NDT80*, n = 56 chromosomes), with maximum-likelihood 2-component Gaussian mixture model (solid lines) and corresponding means (arrows and values). Note that results are indistinguishable for Zip2 (this figure) and Zip3 (text Fig. 4E *bottom left*)

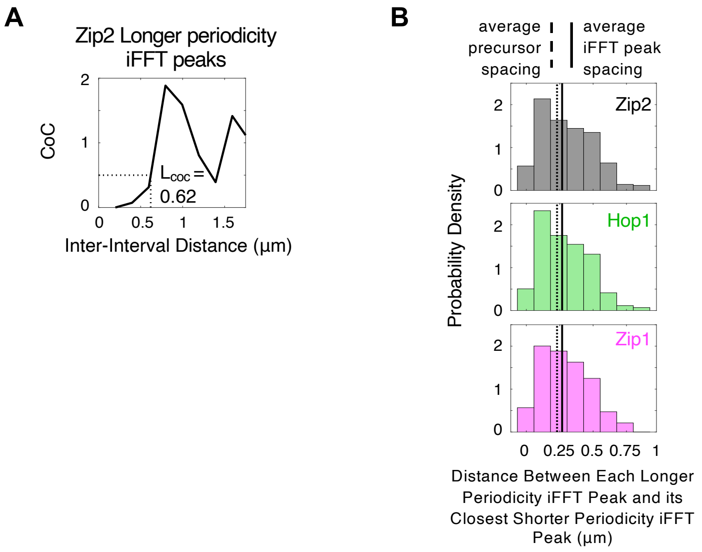

Supplementary Fig. 10. Longer periodicity triad peaks and their relationship to shorter periodicity triad peaks (i.e. canonical crossovers), *extension*. (**A**) Analogous to text Fig. 5A top row. Coefficient of coincidence (CoC) curve for the Zip2 longer periodicity iFFT peaks of pachytene chromosomes isolated from a wild type strain in the absence of pachytene arrest (*NDT80*), with corresponding L_{coc} value (n = 56 chromosomes). (**B**) Analogous to text Fig. 5C. The median distance between each longer periodicity iFFT peak and its nearest shorter periodicity iFFT peak (solid vertical lines; n = 56) matches the expected distance between adjacent early recombination (pre-crossover) precursors (0.23 µm; dashed lines; text), suggesting that both types of triads arise along this same basic array of early recombination precursor sites. Note that results are indistinguishable for Zip2 (this figure) and Zip3 (text Fig. 5A top left; text Fig. 5C top)

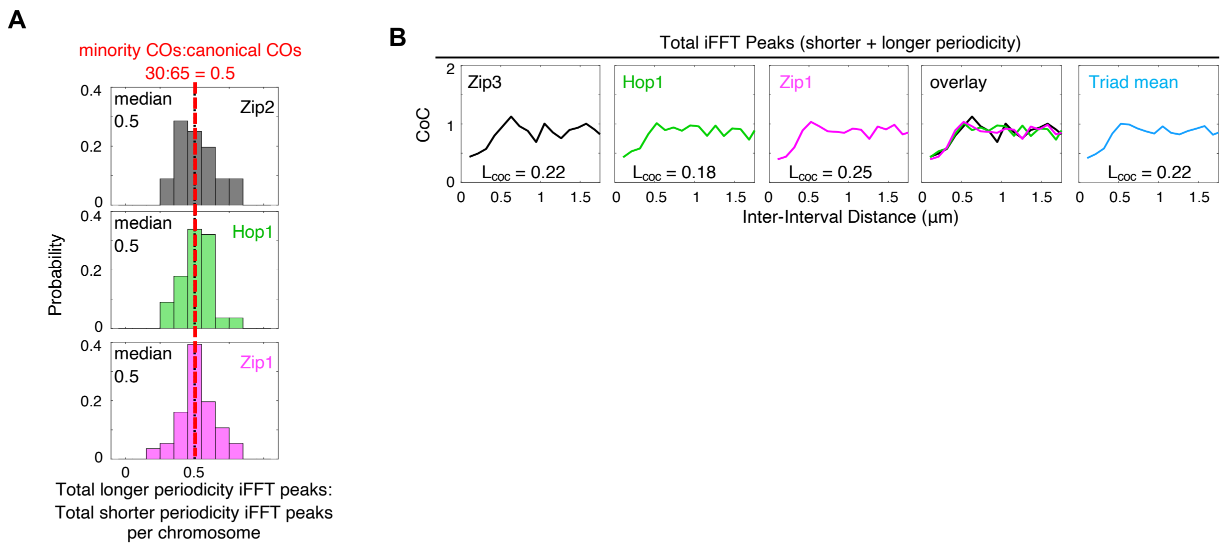

Supplementary Fig. 11. Two lines of evidence that longer periodicity triads arise at the sites of "minority" crossovers, *extended*. (**A**) The ratio of total "minority" crossovers to total "canonical" crossovers is ~0.46:1 (top, *see text*). The median ratios of total longer periodicity iFFT peaks to total shorter periodicity Zip2, Hop1 and Zip1 iFFT peaks detected along pachytene chromosomes isolated from a wild type strain in the absence of pachytene arrest (*NDT80*), is the same as for minority to canonical crossovers (average for all three components taken together = 0.50:1; red dashed line; n = 56 chromosomes). (**B**) CoC curves for total iFFT-defined peaks (shorter and longer periodicities considered together) of each individual triad component (Zip2, Hop1 and Zip1), as well as the mean CoC curve for all three components ('Triad mean'), on wild type chromosomes that were not arrested at pachytene (*NDT80*, n = 56 chromosomes). Note that all results are indistinguishable for Zip3 and Zip2 (compare with text Figs. 6A and 6C).

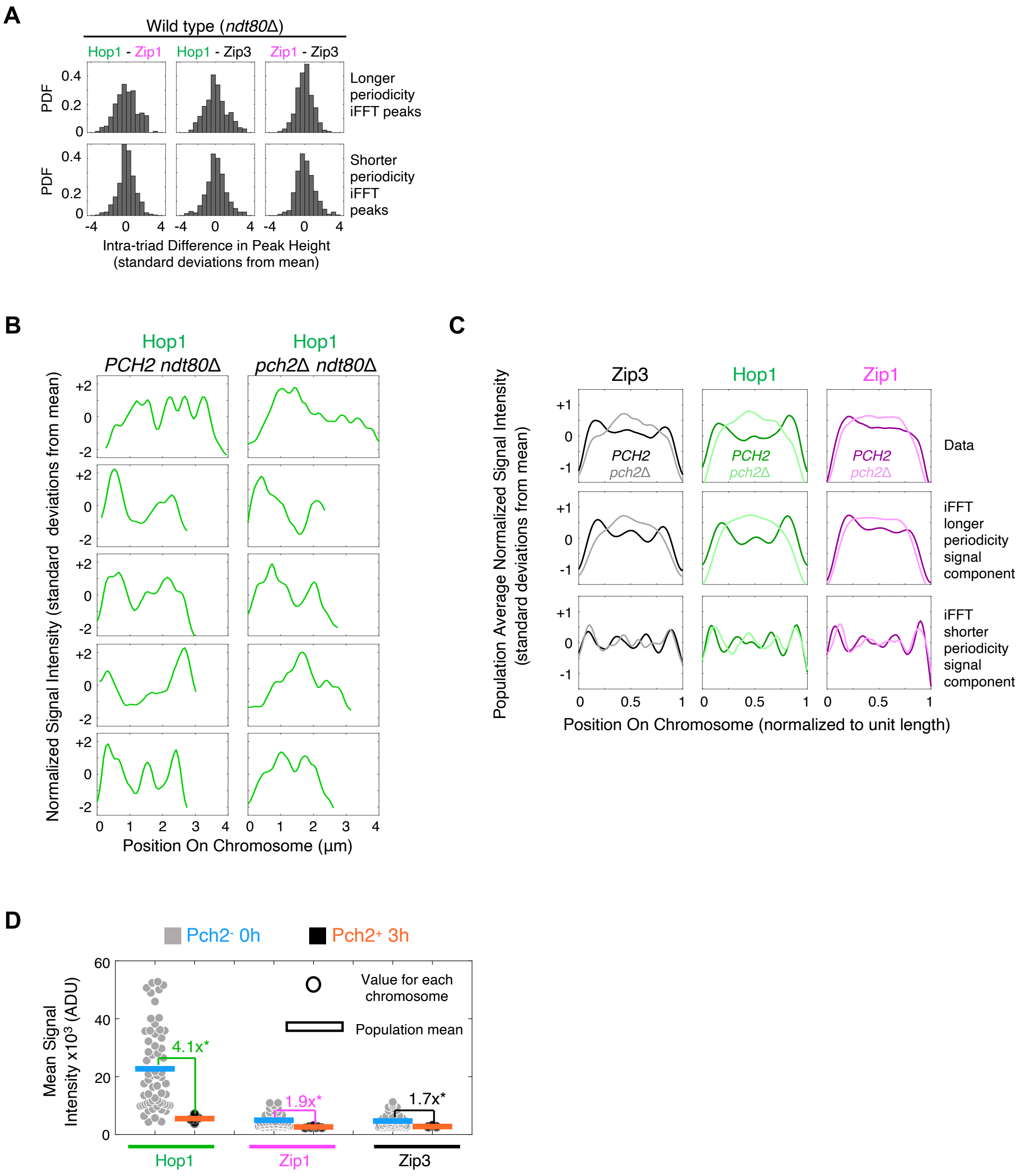

Supplementary Fig. 12. Pch2 modulates the relative and absolute abundances of different triad components, specifically in longer periodicity triads, *extension.* (**A**) Distributions of differences in signal intensities (peak heights) for different pairs of triad components in longer and shorter periodicity triads in wild type meiosis (n = 81 chromosomes), showing no tendency for correlation or anticorrelation. (**B**) Normalized Hop1 signal intensity profiles of five *PCH2 ndt80*Δ and five *pch2*Δ *ndt80*Δ example chromosomes. Some chromosomes show abundant Hop1 loading near a chromosome end, but others do not. (**C**) Normalized signal intensity profiles of *PCH2 ndt80*Δ and *pch2*Δ *ndt80*Δ chromosomes averaged over all measured

chromosomes (n = 81 and 52 chromosomes, respectively). A tendency for Hop1 loading at chromosome ends in wild type meiosis emerges in the average distributions for all chromosomes and is seen for both shorter and longer periodicity iFFT signals. However, absence of Pch2 results in a reduction in near-terminal loading specifically in longer periodicity iFFT signals, not only for Hop1 but also for Zip3 and, perhaps, Zip1. (**D**) Distribution of per chromosome average signal intensities (a proxy for total amount of chromosome-bound protein) and its corresponding population means, for Hop1, Zip1 and Zip3 along pachytene chromosomes isolated from inducible Pch2 cells grown in either the absence of Pch2 expression ($Pch2^-$ 0h; n = 61 chromosomes), or with 3h of Pch2 expression at pachytene ($Pch2^+$ 3h; n = 8 chromosomes). The observed decreases in signal intensity in response to Pch2 expression are statistically significant for all three molecules (p = 0.001, 0.005, and 0.022 for Hop1, Zip1 and Zip3 respectively; two sided 2-sample t-test). Induction of Pch2 expression results in a reduction of the levels of total chromosome-bound Hop1, Zip1 and Zip3, with the biggest effect on Hop1 and similar, lower effects on Zip1 and Zip3. Values indicate the fold-change in population means between $Pch2^+$ conditions (a mimic of wild type), and $Pch2^-$ conditions (a mimic of *pch2*Δ).

Supplementary Note

Which resolvases are involved in maturation of minority crossovers in wild type meiosis?
Current dogma holds that, in wild type meiosis, canonical crossovers are resolved by MutLγ while minority crossovers are resolved by Mus81/Mms4. The notion that minority crossovers are resolved by Mus81/Mms4 arose from the finding that this complex is required for crossover formation in *mutSγ* mutants and, analogously, in *Schizosaccharomyces pombe*, where MutSγ is absent. However, the recombination events that occurring *mutSγ* mutants may be aberrant/unregulated, and thus not the necessarily the same as minority events in wild type meiosis (text). As a result, the question of how minority crossovers are resolved merits further consideration. Specifically, the current results (text) suggest that minority crossovers may require ZMM proteins, previously thought to be specific to canonical crossovers, raising the possibility that minority crossovers might also be resolved by the same resolvase as canonical crossovers, i.e. MutLγ.

Three studies address this issue.

- In budding yeast, *mlh1* and *mms4* mutations both reduce the level of crossovers, with additive effects. However, as stated, the relative contributions of the two resolvases to wild type meiosis cannot be determined because absence of one type of resolvase may allow unscheduled binding and action of the other[4]. Nonetheless, neither mutation alters crossover interference[5, 6]. This finding implies that the two resolvases must be acting equivalently on both canonical and minority crossovers, for the following reason. (i) Both mutations affect the maturation of recombination intermediates and thus act after crossover site positions have been established. (ii) Correspondingly, maturation defects do not affect interference[7, 8]. (iii) Canonical and minority crossovers contribute differently to the interference patterns observed in wild type (text). Thus, if either resolvase acted differentially on one type of crossover versus the other, both single mutants should have exhibited altered interference patterns (e.g. as argued for *mus81* in Arabidopsis, below).

- In Arabidopsis, absence of Mus81 confers a very modest decrease in crossover number plus an apparent increase in crossover interference[9]. This is consistent with differential subtraction of minority crossovers, whose presence will reduce interference as compared to canonical crossovers (text). However, this effect has thus far been defined only for one pair of intervals.

- In tomato, a minority subset of late recombination nodules that lack MLH1 staining can be correlated with minority crossovers[10] (text). However, it remains to be determined whether these events are resolved by MUS81 or are resolved by MLH1 that is present at a cytologically undetectable level.

Supplementary References

1. Agarwal S, Roeder GS. Zip3 provides a link between recombination enzymes and synaptonemal complex proteins. *Cell* **102**, 245-255 (2000).

2. Woglar A, Villeneuve AM. Dynamic Architecture of DNA Repair Complexes and the Synaptonemal Complex at Sites of Meiotic Recombination. *Cell* **173**, 1678-1691 e1616 (2018).

3. Zhang L, Wang S, Yin S, Hong S, Kim KP, Kleckner N. Topoisomerase II mediates meiotic crossover interference. *Nature* **511**, 551-556 (2014).

4. Zakharyevich K, Tang S, Ma Y, Hunter N. Delineation of joint molecule resolution pathways in meiosis identifies a crossover-specific resolvase. *Cell* **149**, 334-347 (2012).

5. de los Santos T, Hunter N, Lee C, Larkin B, Loidl J, Hollingsworth NM. The Mus81/Mms4 endonuclease acts independently of double-Holliday junction resolution to promote a distinct subset of crossovers during meiosis in budding yeast. *Genetics* **164**, 81-94 (2003).

6. Argueso JL, Wanat J, Gemici Z, Alani E. Competing crossover pathways act during meiosis in Saccharomyces cerevisiae. *Genetics* **168**, 1805-1816 (2004).

7. Wang S, Kleckner N, Zhang L. Crossover maturation inefficiency and aneuploidy in human female meiosis. *Cell Cycle* **16**, 1017-1019 (2017).

8. Zhang L, Liang Z, Hutchinson J, Kleckner N. Crossover patterning by the beam-film model: analysis and implications. *PLoS Genet* **10**, e1004042 (2014).

9. Berchowitz LE, Francis KE, Bey AL, Copenhaver GP. The role of AtMUS81 in interference-insensitive crossovers in A. thaliana. *PLoS Genet* **3**, e132 (2007).

10. Anderson LK*, et al.* Combined fluorescent and electron microscopic imaging unveils the specific properties of two classes of meiotic crossovers. *Proc Natl Acad Sci U S A* **111**, 13415-13420 (2014).
