# Supplementary material for: Crossover interference mediates multiscale patterning along meiotic chromosomes
Source: Nat Commun. 2025 Nov 25;16:10453. doi: 10.1038/s41467-025-65423-6 (PMC12647850; doi:10.1038/s41467-025-65423-6)
Supplement: Supplementary file 2 — Reporting Summary [file 41467_2025_65423_MOESM2_ESM.pdf]

nature portfolio

Corresponding author(s): Nancy Kleckner

Last updated by author(s): Aug 20, 2025

Reporting Summary

Nature Portfolio wishes to improve the reproducibility of the work that we publish. This form provides structure for consistency and transparency in reporting. For further information on Nature Portfolio policies, see our Editorial Policies and the Editorial Policy Checklist.

Statistics

For all statistical analyses, confirm that the following items are present in the figure legend, table legend, main text, or Methods section.

n/a	Confirmed	
☐	☒	The exact sample size (*n*) for each experimental group/condition, given as a discrete number and unit of measurement
☐	☒	A statement on whether measurements were taken from distinct samples or whether the same sample was measured repeatedly
☐	☒	The statistical test(s) used AND whether they are one- or two-sided<br>*Only common tests should be described solely by name; describe more complex techniques in the Methods section.*
☒	☐	A description of all covariates tested
☒	☐	A description of any assumptions or corrections, such as tests of normality and adjustment for multiple comparisons
☐	☒	A full description of the statistical parameters including central tendency (e.g. means) or other basic estimates (e.g. regression coefficient) AND variation (e.g. standard deviation) or associated estimates of uncertainty (e.g. confidence intervals)
☐	☒	For null hypothesis testing, the test statistic (e.g. *F*, *t*, *r*) with confidence intervals, effect sizes, degrees of freedom and *P* value noted<br>*Give P values as exact values whenever suitable.*
☒	☐	For Bayesian analysis, information on the choice of priors and Markov chain Monte Carlo settings
☒	☐	For hierarchical and complex designs, identification of the appropriate level for tests and full reporting of outcomes
☒	☐	Estimates of effect sizes (e.g. Cohen's *d*, Pearson's *r*), indicating how they were calculated

Our web collection on statistics for biologists contains articles on many of the points above.

Software and code

Policy information about availability of computer code

Data collection	Signal intensity profiles of pachytene chromosomes were extracted from acquired images using open-source software FIJI (ImageJ 1.54f)
Data analysis	Signal intensity profiles of pachytene chromosomes were analyzed using MATLAB software. Custom code is freely available on GitHub: https://github.com/mwhite4/multiscaleCrossoverPatterning (doi.org/10.5281/zenodo.16920249). For summing all Fourier transforms (Fig. 2D, E and Supplementary Fig. 6), the discrete Fourier transform of each experimental normalized signal intensity profile was calculated using Mathematica (command 'Fourier'). The relevant Mathematica code is also available from https://github.com/mwhite4/multiscaleCrossoverPatterning (doi.org/10.5281/zenodo.16920249).

For manuscripts utilizing custom algorithms or software that are central to the research but not yet described in published literature, software must be made available to editors and reviewers. We strongly encourage code deposition in a community repository (e.g. GitHub). See the Nature Portfolio guidelines for submitting code & software for further information.

Data

Policy information about availability of data

All manuscripts must include a data availability statement. This statement should provide the following information, where applicable:

- Accession codes, unique identifiers, or web links for publicly available datasets
- A description of any restrictions on data availability
- For clinical datasets or third party data, please ensure that the statement adheres to our policy

Primary image files and signal intensity profiles of traced chromosomes have been uploaded to Harvard Dataverse (https://dataverse.harvard.edu; dataset "Crossover Interference Mediates Multiscale Patterning Along Meiotic Chromosomes") and and has been published with a cited DOI (https://doi.org/10.7910/DVN/5LEWYF)

Research involving human participants, their data, or biological material

Policy information about studies with human participants or human data. See also policy information about sex, gender (identity/presentation), and sexual orientation and race, ethnicity and racism.

Reporting on sex and gender	This study did not involve human participants, their data, or biological material.
Reporting on race, ethnicity, or other socially relevant groupings	This study did not involve human participants, their data, or biological material.
Population characteristics	This study did not involve human participants, their data, or biological material.
Recruitment	This study did not involve human participants, their data, or biological material.
Ethics oversight	This study did not involve human participants, their data, or biological material.

Note that full information on the approval of the study protocol must also be provided in the manuscript.

Field-specific reporting

Please select the one below that is the best fit for your research. If you are not sure, read the appropriate sections before making your selection.

☒ Life sciences ☐ Behavioural & social sciences ☐ Ecological, evolutionary & environmental sciences

For a reference copy of the document with all sections, see nature.com/documents/nr-reporting-summary-flat.pdf

Life sciences study design

All studies must disclose on these points even when the disclosure is negative.

Sample size	Statistical methods were not used to determine sample size prior to analysis and were determined based on practical limitations of isolating and tracing yeast meiotic prophase chromosomes. Sample sizes were deemed sufficient due to the close correspondence of quantitative results with previously published studies (e.g. Fig. 4B, D, E).
Data exclusions	No data were excluded from the analyses.
Replication	Experiments were performed at least three times independently. All attempts at replication were successful. Samples for 'wild-type' ndt80D and NDT80 datasets were acquired on separate dates, by separate co-authors, and high correspondence of Zip1 and Hop1 patterns and Zip2/Zip3 patterns were achieved (e.g. Fig. 3C - F versus Supplementary Fig. 5B - E).
Randomization	Chromosomes were allocated into their respective group based on the genotype of the cells from which they were isolated. Randomization is not relevant to this study since groups are composed of clonal populations of cells.
Blinding	The investigators were not blinded to group allocation. Blinding was not relevant to this study as the same automated computational analysis pipeline was used for all groups.

Reporting for specific materials, systems and methods

We require information from authors about some types of materials, experimental systems and methods used in many studies. Here, indicate whether each material, system or method listed is relevant to your study. If you are not sure if a list item applies to your research, read the appropriate section before selecting a response.

Materials & experimental systems

n/a	Involved in the study
☐	☒ Antibodies
☒	☐ Eukaryotic cell lines
☒	☐ Palaeontology and archaeology
☒	☐ Animals and other organisms
☒	☐ Clinical data
☒	☐ Dual use research of concern
☒	☐ Plants

Methods

n/a	Involved in the study
☒	☐ ChIP-seq
☒	☐ Flow cytometry
☒	☐ MRI-based neuroimaging

Antibodies

Antibodies used

Primary antibodies
mouse monoclonal anti-Myc (Santa Cruz Biotechnology; catalog# sc-40; lot# H1721)
goat polyclonal anti-Zip1 (Santa Cruz Biotechnology; catalog# sc-15632)
rabbit polyclonal anti-Hop1 (gift from Franz Klein, Max Perutz Labs, Vienna)
rat polyclonal anti-Zip2 (Abclonal; custom antibody, project# AP20721)

Secondary antibodies
donkey anti-mouse IgG-AF555 (Invitrogen; catalog# A-31570; lot# 1117032)
donkey anti-rabbit IgG-AF488 (Invitrogen; catalog# A-21206; lot# 2289872)
donkey anti-goat IgG-AF647 (Invitrogen; catalog# A-21447; lot# 1739289)
donkey anti-rat IgG-AF488 (Invitrogen; catalog# A-21208; lot# 2310102)
donkey anti-rabbit IgG-AF555 (Invitrogen; catalog# A-31572)

Antibodies were used at a dilution of 1/1000 in 1xTBS-1% BSA

Validation

The following primary antibodies have been validated in published studies prior to this work. Their staining patterns were confirmed by us to match known localization patterns of the respective proteins on yeast pachytene chromosomes.
- mouse monoclonal anti-Myc: Zhang L, Wang S, Yin S, Hong S, Kim KP, Kleckner N. Topoisomerase II mediates meiotic crossover interference. Nature 511, 551-556 (2014).
- goat polyclonal anti-Zip1: Zhang L, Wang S, Yin S, Hong S, Kim KP, Kleckner N. Topoisomerase II mediates meiotic crossover interference. Nature 511, 551-556 (2014).
- rabbit polyclonal anti-Hop1: Borner GV, Barot A, Kleckner N. Yeast Pch2 promotes domainal axis organization, timely recombination progression, and arrest of defective recombinosomes during meiosis. Proc Natl Acad Sci U S A 105, 3327-3332 (2008).

For primary antibody rat anti-Zip2, antiserum ELISA test data showed that post 4th immunization antisera were positive against antigen protein strongly (Abclonal); western blots against recombinant antigen using purified antibodies showed single bands that were consistent with the expected molecular weight, that 5 ng of antigen can be detected by antibodies at a dilution of 1:1000 and that the concentration of antibodies fall within the normal range (Abclonal). Here, we further validate this antibody by demonstrating the close correspondence of detected immunofluorescence patterns of Zip2 and its partner protein Zip3 (Supplemental Fig. 1).

Plants

Seed stocks

No plants were used in this study.

Novel plant genotypes

No plants were used in this study.

Authentication

No plants were used in this study.
